# The development of ingroup favoritism in repeated social dilemmas

**DOI:** 10.3389/fpsyg.2015.00476

**Published:** 2015-04-28

**Authors:** Angela R. Dorrough, Andreas Glöckner, Dshamilja M. Hellmann, Irena Ebert

**Affiliations:** ^1^Department of Psychology, Max Planck Institute for Research on Collective GoodsBonn, Germany; ^2^Department of Social Psychology, University of SiegenSiegen, Germany; ^3^Department of Psychology, University of GöttingenGöttingen, Germany; ^4^Faculty of Psychology, University of Koblenz-LandauLandau, Germany

**Keywords:** ingroup favoritism, intergroup contact, prisoner's dilemma, social identity, social dilemmas

## Abstract

In two comprehensive and fully incentivized studies, we investigate the development of ingroup favoritism as one of two aspects of parochial altruism in repeated social dilemmas. Specifically, we test whether ingroup favoritism is a fixed phenomenon that can be observed from the very beginning and remains stable over time, or whether it develops (increases vs. decreases) during repeated contact. Ingroup favoritism is assessed through cooperation behavior in a repeated continuous prisoner's dilemma where participants sequentially interact with 10 members of the ingroup (own city and university) and subsequently with 10 members of the outgroup (other city and university), or vice versa. In none of the experiments do we observe initial differences in cooperation behavior for interaction partners from the ingroup, as compared to outgroup, and we only observe small differences in expectations regarding the interaction partners' cooperation behavior. After repeated interaction, however, including a change of groups, clear ingroup favoritism can be observed. Instead of being due to gradual and potentially biased updating of expectations, we found that these emerging differences were mainly driven by the change of interaction partners' group membership that occurred after round 10. This indicates that in social dilemma settings ingroup favoritism is to some degree dynamic in that it is enhanced and sometimes only observable if group membership is activated by thinking about both the interaction with the ingroup and the outgroup.

## Introduction

Cooperation is an essential prerequisite for human social life, but it often involves social dilemma situations that require individuals to decide whether to maximize selfish or collective interests. A typical social dilemma situation is the following: a team of two people works together on a collectively profitable project where benefits are shared evenly and independently of individual contributions. Although the collective benefit would be highest if both team members contributed as much as possible, the benefit of each individual is even higher if one chooses the non-cooperative option given that the other member cooperates.

Numerous factors have been shown to influence the tendency of individuals to behave cooperatively in social dilemmas or not (for overviews, see Dawes, 1980; Komorita and Parks, 1995; Zelmer, 2003; Van Lange et al., 2013). One important determinant is group affiliation, that is, whether the partner is perceived as a member of the ingroup or the outgroup. Studies have repeatedly demonstrated ingroup favoritism, that is, the tendency to favor members of one's ingroup over outgroup members both in social dilemma tasks (e.g., Goette et al., 2006; Simpson, 2006; Balliet et al., 2014; De Dreu et al., 2014) and beyond (e.g., for helping behavior in violent situations: Levine et al., 2006; or after natural disasters: Levine and Thompson, 2004; see Hewstone et al., 2002, for an overview).

In the context of social dilemmas, ingroup favoritism is usually found in the form of higher cooperation rates toward ingroup members compared to outgroup members (e.g., Wit and Wilke, 1992; De Cremer and van Vugt, 1999; Goette et al., 2006, 2012) and higher expectations regarding cooperation behavior for the ingroup as compared to the outgroup (e.g., Yamagishi et al., 2008). As applied to the introductory example above, each individual's tendency to contribute to the joint project should be higher if the interaction partner belongs to the same group as compared to a different group (e.g., the same vs. a different university).

Overall, a comprehensive meta-analysis summarizing the results of 212 studies from 77 publications (Balliet et al., 2014) finds a small to medium effect size, indicating that people are more cooperative with ingroup compared to outgroup members (*d* = 0.32), and a slightly stronger effect on expectations concerning cooperation (*d* = 0.41). The meta-analysis identified several moderators for ingroup favoritism, such as bilateral knowledge of group membership (i.e., both people know whether they are from the same or different groups) or the frequency of interactions (one shot vs. repeated). Although, most of the studies assessing repeated interactions acknowledge that cooperation in general changes over time (typical declining pattern of cooperation), none of the studies considers changes in ingroup favoritism over repeated contact.

In the current work, we therefore aim to investigate possible dynamics of ingroup favoritism at a cognitive and a behavioral level over repeated interactions.

### Dynamic aspects of ingroup favoritism

The theory of parochial altruism explains ingroup favoritism from an evolutionary perspective. It states that increased cooperativeness toward the ingroup (ingroup love) is due to parochial altruistic norms, which have an evolutionary origin. According to the theory, ingroup favoritism represents, together with aggressiveness against the outgroup (outgroup hate), a genetic or cultural trait that has co-evolved in humans (Bernhard et al., 2006; Rusch, 2014). Following this rationale, it could be assumed that ingroup favoritism might prevail from the first of repeated interactions. Several models for behavior in social dilemmas (Van Lange et al., 2013) would predict the same. According to the goal expectation theory proposed by Pruitt and Kimmel (1977), individuals cooperate if they adopt the goal of cooperation and expect their partner to reciprocate. Similarly, Bogaert et al. (2008) proposed in their model that cooperation is driven by an integration of context-specific cooperative goals and context-specific expectations. Joint group membership can function as an important context cue that influences both the likelihood for adopting cooperation goals and the expectations that the interaction partner will reciprocate cooperation. The latter class of models, however, also highlights the fact that repeated experiences can at least change expectations and potentially also the tendency of individuals to take over cooperation goals. Experiences should be updated in a roughly rational manner, in that expectations after some time reflect the average behavior of ingroup and outgroup members in the real world.

As stated in the contact hypothesis (Allport, 1954), repeated interactions with other persons (e.g., members from the outgroup) can reduce prejudice and might therefore also reduce differences between ingroup and outgroup. Indeed, previous research has shown that discrimination against members from the outgroup is reduced after repeated contact (Birtel and Crisp, 2012; see Pettigrew and Tropp, 2006, for a meta-analysis). Not surprisingly, and also in line with a rational updating of expectations, the opposite effect (i.e., increased discrimination) was found in cases where the interaction with the outgroup included negative experiences (Barlow et al., 2012). Following this line of reasoning and in line with the models explained above, both a reduction and an enhancement of ingroup favoritism are conceivable, depending on the actual experiences made with different group members. Expectations and cooperation behavior should be adjusted, in line with actual experienced cooperation and independently of group affiliation. If one experiences higher cooperation from ingroup members than from outgroup members, own expectations and cooperation should be adjusted accordingly and ingroup favoritism should increase (or emerge if it does not exist from the beginning). However, if both the ingroup and the outgroup cooperate to the same degree, ingroup favoritism should disappear, given an unbiased adjustment of expectations and cooperation.

In contrast, one can assume that the adjustment of expectations and cooperation behavior in repeated interactions is not completely rational, depending not only on the degree of experienced cooperativeness. Rather, it is possible that the group affiliation of the interaction partner is a key factor for the assessment of her or his behavior and the adjustment of own expectations and cooperation behavior in subsequent interactions. Categorical thinking about ingroup and outgroup can shape the perception of the behavior of others (Macrae and Bodenhausen, 2000; Mussweiler and Ockenfels, 2013), which results in different attribution patterns in explaining the behavior of the ingroup and the outgroup member. The ultimate attribution error states that negative behavior is attributed dispositionally when it is shown by the outgroup, whereas positive behavior is attributed externally, and vice versa, for the ingroup (Pettigrew, 1979). According to social identity theory, this reflects the need to develop and maintain a positive self-concept by maximizing the positive distinctiveness of the ingroup in contrast to an outgroup (Tajfel and Turner, 1979; Hewstone et al., 2002). As a consequence, a cognitive mechanism that facilitates the processing of incoming social information in an ingroup favoring light might be activated, causing different generalization patterns for behavior from ingroup as compared to outgroup members (Henderson-King and Nisbett, 1996). If patterns of observations are consistently attributed in a rather “friendly” manner for interactions with the ingroup and in an “unfriendly” manner for the outgroup, the (objectively) same experiences of behavior should be generalized quite differently. Based on the important work on the ultimate attribution error and differential generalization patterns, people could be expected to show an ingroup-favoring generalization bias in repeated social dilemma interactions, as follows: while positive behavior (cooperation) is more strongly generalized from one to subsequent members of the ingroup, negative behavior is more strongly generalized to outgroup members. In contrast to the prediction of rational updating of expectations, this ingroup-favoring generalization bias would lead to increasing ingroup favoritism.

In two experiments we investigate (i) whether ingroup favoritism is mainly driven by fixed initial differences or dynamics that develop over repeated interactions, and (ii) whether these dynamics reflect rational updating or an ingroup-favoring generalization bias.

Investigating initial differences and potential dynamics of ingroup favoritism is methodologically demanding. To assure high internal and external validity we decided to use a repeated version of a social dilemma game, in which participants interact with different members of both groups (i.e., stranger matching with change of groups after half of the trials). Additionally to avoid effects due to artificial responses, participants interact with real interaction partners and we use real incentives. Also we use relatively salient and to some degree natural groups. Furthermore, to learn more about drivers for possible dynamics, expectations regarding cooperation are repeatedly measured.

Interestingly, although there are many studies published on ingroup favoritism in social dilemmas (Balliet et al., 2014, for an overview), none of them can be directly used to derive clear predictions concerning our research questions. First, none of them fulfills all the above mentioned characteristics to properly investigate dynamics at the same time. Specifically, from the seven studies reporting results from repeated interactions in prisoner's dilemmas, four do involve fake interaction partners, which makes potential conclusions concerning dynamics questionable (Wrightsman et al., 1972; Baxter, 1973; Dion, 1973; Parks et al., 2001). Of the remaining studies two do not involve interactions with members from both groups (Wilson and Kayatani, 1968; Wallace and Rothaus, 1969) and another one does not use real groups but minimal groups instead (Wilson et al., 1965). Second, due to being interested in different topics, most studies do not report analyses concerning the dynamics of ingroup and outgroup cooperation over repeated interactions. An exception is the study by Wallace and Rothaus (1969), which shows relatively stable cooperation over time in the ingroup condition, but a decrease in cooperation in the outgroup condition. However, the study does not report changes in ingroup favoritism (comparison between ingroup and outgroup cooperation) and, as stated before, it does not include alternating interaction partners nor interactions with both groups. Considering these limitations and taking into account that most relevant studies have been published more than 40 years ago our research questions cannot be answered based on published results and neither by re-analyzing existing data. Therefore, we conducted two new studies to directly address them.

### Overview of the experiments

In our experiments, we investigate ingroup favoritism at a cognitive and a behavioral level by measuring (a) whether there are higher expectations regarding cooperation behavior for the ingroup as compared to the outgroup and (b) whether there is higher cooperation toward the ingroup compared to the outgroup. We used repeated interactions in a prisoner's dilemma with a stranger-rematching protocol, in which individuals knew that they would never interact with the same partner twice. Participants sequentially interacted (got into contact) with 10 different members from the ingroup and subsequently with 10 different members from the outgroup, or vice versa, which constituted a group change manipulation (contact with both groups) between the two parts of the experiment. Group salience was induced by a Skype conference of about 2 min at the beginning of the experiment, during which participants could confirm that the outgroup actually existed. Participants sitting together with their ingroup in one experimental laboratory (in separate cubicles) could see the outgroup's laboratory, but could not identify the individual participants. Besides the Skype conference, participants in Experiment 1 knew that they would play the prisoner's dilemma game with different people from their own university and city (ingroup) or another university and city (outgroup). To enforce the salience of the ingroup-outgroup differentiation, and to make sure that our manipulation did not prime the common identity of being a student, we conducted Experiment 2 (which also served as a partial replication of Experiment 1), in which participants were additionally told which university and city their interaction partners came from.

On theoretical ground, we investigate the development of ingroup favoritism and the underlying process driving this development. In order to do so, we test for differential hypotheses concerning generalization patterns that follow from different classes of models and examine whether the dynamic development of ingroup favoritism reflects rational updating or an ingroup-favoring generalization bias.

## Experiment 1

Experiment 1 assesses whether ingroup favoritism is a fixed phenomenon that can be observed from the first interaction onwards and that remains stable over time, or whether it is a dynamic construct that develops over time. Besides, Experiment 1 aims to identify drivers for potential dynamics in ingroup favoritism.

### Methods

#### Participants and design

Seventy-two people (mainly students at the University of Bonn and the University of Erfurt, 44 of whom were female) were recruited via the online recruitment tool *Orsee* (Greiner, 2004) and took part in the experiment. Subjects participated in continuous prisoner's dilemma games (for a detailed description, see below) in groups of two. We manipulated as within-subjects factor whether individuals played with different individuals from their own city and university (ingroup) or with different individuals from another city and university (outgroup). Sessions consisted of 24 individuals, 12 in the experimental laboratory in Bonn and 12 in the experimental laboratory of the University of Erfurt. Assignment to dyads and conditions was anonymous and random. Participants played 10 rounds with different people from one group. After round 10, a group change took place, followed by another 10 rounds with different members of the other group. For both parts, we used a stranger-rematching protocol, so that participants never interacted with the same person twice. The sequence of ingroup and outgroup conditions was counterbalanced (session-wise).^1^ The experiment was computerized and run using *Bonn Experimental System* (BoXS, Seithe, 2012). Completing the experiment took participants about 60 min overall. Participants' payments depended on their decisions, and earnings ranged from 8.30 to 23.90 Euros (approx. USD 11.20 to USD 32.30).^2^

#### Materials and procedure

All participants were informed about the structure of the game by detailed instructions which they read before beginning with the experiment. Communication was forbidden throughout the experiment. After reading the instructions, participants answered six control questions to assure they had understood the rules. Answers were checked by the experimenters and questions were answered individually. After participants had read the instructions and answered the control questions, we arranged a live Skype conference with the other lab to assure our participants that they would interact with people from another lab in real time and to make group affiliation salient. As a manipulation check for the effectiveness of our ingroup–outgroup manipulation, before starting with the prisoner's dilemma game, we assessed perceived interpersonal closeness with both groups using a pictorial scale containing seven graphical items for the overlap of self and ingroup as well as self and outgroup (Aron et al., 1992; Schubert and Otten, 2002). The repeated prisoner's dilemma worked as follows: In each round, participants were given a round endowment of 10 Taler (1 Taler = 0.05 Euro). Both players decided simultaneously which amount between 0 and 10 of their round endowment to transfer to their current interaction partner, whereas they kept the rest (10 – transferred amount) in their private account. The money transferred to the interaction partner was multiplied by a factor of 2. If both participants in a dyad transferred their whole endowment of 10 Taler, each player earned 20 Taler in this round. Hence, there was a potential collective gain of 100% that could be realized by transferring Taler to the interaction partner (cooperating). The individual payoff was maximized, however, if a player did free-ride on the cooperation of his or her interaction partner, that is, if the player kept the round endowment and enjoyed the money the other player transferred to her/him. Before participants made their cooperation decisions, they were asked about their expectations regarding their current interaction partner's cooperation by typing any amount between 0 and 10 which they expected to receive from their current partner.^3^ At the end of each round participants were informed about the amount of money their interaction partner transferred to them and their earning in the current round.

### Results

The interpersonal closeness scale reveals that people felt a higher overlap between self and ingroup (*M* = 4.88, *SD* = 1.61), as compared to self and outgroup (*M* = 3.05, *SD* = 1.49), prior to playing the prisoner's dilemma game [*t*_(58)_ = 8.79, *p* < 0.001, *d* = 1.19], indicating that our ingroup–outgroup manipulation was successful and induced a large effect.^4^

First, we examined whether ingroup favoritism prevails overall. We ran two ordinary least square (OLS) regressions over all rounds, which predicted cooperation behavior and expectations by group affiliation of the interaction partner (dummy coded; outgroup = 0 vs. ingroup = 1). For the regressions, we clustered at the individual level (Rogers, 1994) and controlled for counterbalancing condition (ingroup first = 0 vs. outgroup first = 1) and experimental lab (Bonn = 0 vs. Erfurt = 1).^5^ We found significantly increased average cooperation for members of the ingroup as compared to members of the outgroup, *b* = 0.70, *t*_(71)_ = 3.07, *p* = 0.003, *d* = 0.21 (Table 1, Model 1). Hence, overall cooperation toward the ingroup is 0.70 (out of 10) Talers higher for interactions with members from the ingroup as compared to interactions with members from the outgroup. A similar effect is observed for average expectations concerning cooperation, which is increased by about the same magnitude for the ingroup as compared to the outgroup, *b* = 0.74, *t*_(71)_ = 3.00, *p* = 0.004, *d* = 0.24 (Table 1, Model 2).

**Table 1:** **Regression analysis for cooperation and expectations**.

	**(1) Experiment 1 cooperation**	**(2) Experiment 1 expectation**	**(3) Experiment 2 cooperation**	**(4) Experiment 2 expectation**	**(5) Overall cooperation**	**(6) Overall expectation**
Interaction partner's group affiliation (0 = outgroup; 1 = ingroup)	0.701^**^	0.740^**^	1.005^***^	1.146^***^	0.875^***^	0.972^***^
	(3.07)	(3.00)	(4.93)	(5.58)	(5.75)	(6.14)
Experimental lab (0 = Bonn, 1 = Erfurt)	0.490	0.671	0.278	0.110	0.369	0.351
	(0.93)	(1.59)	(0.57)	(0.30)	(1.03)	(1.27)
Counterbalancing condition (ingroup first = 0 vs. outgroup first = 1)	1.534^*^	1.259^**^	0.749	0.731^*^	1.044^**^	0.899^**^
	(2.51)	(2.71)	(1.54)	(2.02)	(2.80)	(3.27)
Constant	2.966^***^	3.323^***^	3.119^***^	3.351^***^	3.039^***^	3.342^***^
	(7.65)	(10.52)	(8.25)	(10.31)	(11.23)	(14.72)
Observations	1440	1440	1920	1920	3360	3360
Subjects/Cluster	72	72	96	96	168	168
Adjusted *R*^2^	0.059	0.061	0.030	0.043	0.039	0.045

t-statistics in parentheses, OLS regression analysis used, standard errors are clustered at the individual level,

* p < 0.05,

** p < 0.01,

*** p < 0.001.

**Figure 1:**
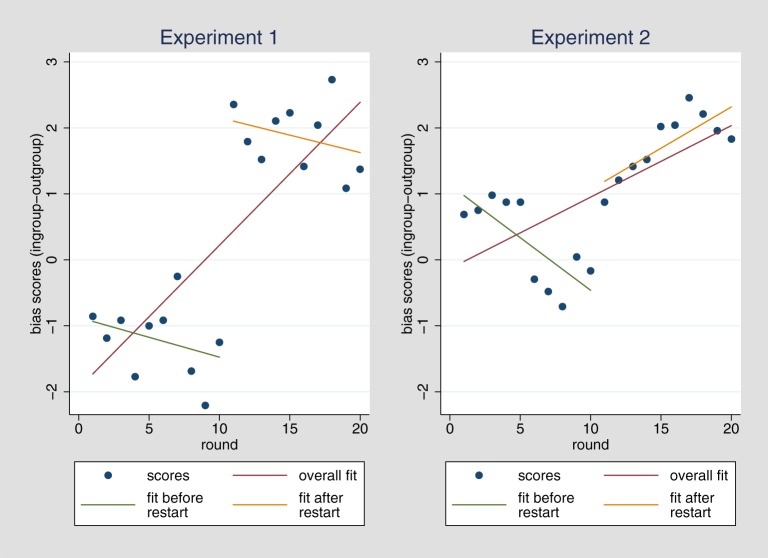
**Development of bias score (ingroup – outgroup cooperation) from round 1 to round 20 with group change and restart after round 10**.

In order to examine whether ingroup favoritism is a fixed phenomenon that remains stable or whether it develops dynamically over repeated contact, we calculated a mean bias score (difference between ingroup and outgroup cooperation) for each round and collapsed over all participants, resulting in 20 data points (one for each round). Running a regression predicting this bias score by the variable round, we found that ingroup favoritism significantly increased over time (rounds), *b* = 0.21, *t*_(18)_ = 5.10, *p* < 0.001, which is in line with the dynamic perspective. Hence, in each of the 20 rounds, favoring the ingroup over the outgroup increased by 0.21 Talers, which is represented by the red regression line in Figure 1 (left).

Interestingly, when comparing average contribution rates for the ingroup and the outgroup, participants did not show any ingroup favoritism (and even a tendency in the opposite direction) in the first round, *b* = −0.85, *t*_(69)_ = −1.05, *p* = 0.298 (Table 2, Model 1), but a tendency to do so for the last round, *b* = 1.38, *t*_(69)_ = 1.94, *p* = 0.056 (Table 2, Model 2).^6^ This results is also illustrated in Figure 1 (left), in that the bias score for the first round (as well as subsequent rounds up to round 10) is negative, whereas it is positive for the last round.^7^

**Table 2:** **Regression analyses for cooperation in the first and the last round**.

**Cooperation (in Taler)**	**(1) Experiment 1 first round**	**(2) Experiment 1 last round**	**(3) Experiment 1 first round**	**(4) Experiment 1 last round**	**(5) Overall first round**	**(6) Overall last round**
Interaction partner's group affiliation (0 = outgroup; 1 = ingroup)	−0.854	1.375^+^	0.687	1.833^**^	0.0486	1.653^***^
	(−1.05)	(1.94)	(0.94)	(2.84)	(0.09)	(3.53)
Experimental lab (0 = Bonn, 1 = Erfurt)	−0.0278	1.361^*^	0.229	0.417	0.119	0.821^+^
	(−0.04)	(2.04)	(0.31)	(0.65)	(0.23)	(1.77)
Constant	6.597^***^	0.819	5.677^***^	1.125^*^	5.996^***^	1.006^**^
	(8.60)	(1.55)	(9.01)	(2.02)	(12.42)	(2.62)
Observations	72	72	96	96	168	168
Adjusted *R*^2^	−0.013	0.077	−0.011	0.064	−0.012	0.075

t-statistics in parentheses, OLS regression analysis used

+ p < 0.10,

* p < 0.05,

** p < 0.01,

*** p < 0.001.

**Figure 2:**
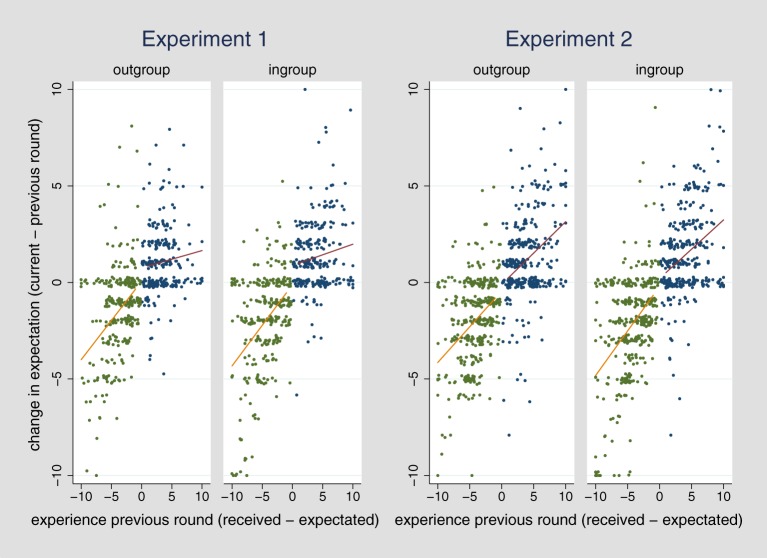
**Experience in previous round (received Taler – expected Taler) dependent on the change in expectation (current – previous round) for the ingroup and the outgroup in Experiment 1 and 2**.

Furthermore, we aimed to investigate what drives the change in ingroup favoritism over time and led to the development of ingroup favoritism after the first round. Since ingroup favoritism concerning cooperation was not observable in the first round, rational updating cannot account for the observed dynamics. We therefore focus the investigation on the ingroup-favoring generalization bias. According to an ingroup-favoring generalization bias, positive experiences should be more strongly generalized over the ingroup, as compared to the outgroup, and vice versa for negative experiences. Stated differently, receiving more than one expects should lead to a stronger increase in expectations about members from the ingroup as compared to the outgroup in the following round. Conversely, receiving less than one expects should lead to a stronger decrease in expectations for the outgroup, as compared to the ingroup. Figure 2 (left) presents the observed changes in expectations concerning cooperation as a function of positive or negative experiences in the previous round.

As indicated by the similar slope of the regression lines for ingroup and outgroup, and as further confirmed by a statistical analysis, no differential effects were observed (interaction between experience and ingroup) for positive, *b* = 0.03, *t*_(70)_ = 0.51, *p* = 0.610 and negative experiences, *b* = −0.01, *t*_(68)_ = −0.09, *p* = 0.93.^8^ Hence, there was no support for an ingroup-favoring generalization bias (Table 3, Models 1 and 2).

**Table 3:** **Regression analysis for changes in expectations due to positive or negative experience in the previous round**.

**Change in expectation (current round – previous round)**	**(1) Experiment 1 positive experiences**	**(2) Experiment 1 negative experiences**	**(3) Experiment 2 positive experiences**	**(4) Experiment 2 negative experiences**	**(5) Overall positive experiences**	**(6) Overall negative experiences**
Experience (amount received – expectation)	0.122^*^	0.411^***^	0.312^***^	0.417^***^	0.237^***^	0.419^***^
	(2.54)	(6.25)	(7.21)	(6.68)	(6.86)	(9.24)
Interaction partner's group affiliation (0 = outgroup; 1 = ingroup)	0.0661	−0.263	0.324	0.210	0.243	−0.0136
	(0.30)	(−0.73)	(1.11)	(0.63)	(1.20)	(−0.05)
Experience*group affiliation	0.0281	−0.0109	−0.0239	0.0899	−0.0148	0.0433
	(0.51)	(−0.09)	(−0.32)	(0.93)	(−0.28)	(0.56)
Experimental lab (0 = Bonn, 1 = Erfurt)	0.307	0.367	0.00204	−0.232	0.163	0.0508
	(1.51)	(1.48)	(0.01)	(−0.89)	(1.05)	(0.28)
Counterbalancing condition	−0.191	0.0396	−0.0886	0.182	−0.172	0.0619
(ingroup first = 0 vs. outgroup first = 1)	(−0.88)	(0.14)	(−0.42)	(0.72)	(−1.12)	(0.34)
Constant	0.621^**^	−0.364	0.0176	−0.415^+^	0.273^+^	−0.341^*^
	(3.19)	(−1.64)	(0.08)	(−1.78)	(1.67)	(−2.11)
Observations	524	606	691	751	1215	1357
Subjects/Cluster	71	69	95	95	166	164
Adjusted *R*^2^	0.022	0.170	0.118	0.169	0.076	0.169

t-statistics in parentheses, OLS regression analysis used, standard errors are clustered at the individual level; variables group affiliation and experience centered; positive (negative) experience take into account only cases in which people received more (less) than they expected;

+ p < 0.10,

* p < 0.05,

** p < 0.01,

*** p < 0.001.

For exploratory reasons, we further investigate whether changing groups had any effect, which might activate social identity by making group membership more salient. Comparing cooperation rates between round 10 (last round with one group) and 11 (first round with the other group), there is indeed a sharp increase in ingroup favoritism, as indicated by a significant interaction between round (round 10 vs. 11) and ingroup^9^, (Table 4, Model 1).

**Table 4:** **Regression analysis for changes in cooperation due to group change from round 10 to round 11**.

**Cooperation (in Taler)**	**(1) Experiment 1**	**(2) Experiment 1**	**(3) Experiment 2**	**(4) Experiment 2**	**(5) Overall**	**(6) Overall**
Interaction partner's group affiliation	0.552	0.255	0.354	−0.160	0.493^+^	−0.0193
(0 = outgroup; 1 = ingroup)	(1.18)	(0.96)	(0.94)	(−0.54)	(1.73)	(−0.09)
Round (round 10 = 0, round 11= 1)	1.573^**^	0.461^+^	2.250^***^	0.898^*^	1.951^***^	0.614^**^
	(3.37)	(1.69)	(6.00)	(2.57)	(6.84)	(2.68)
Group affiliation^*^round	3.604^*^	0.342	1.042	0.140	1.924^*^	0.359
	(2.46)	(0.43)	(0.88)	(0.14)	(2.16)	(0.52)
Experimental lab (0 = Bonn, 1 = Erfurt)	0.139	−0.0341	−0.167	0.144	−0.0357	0.114
	(0.22)	(−0.10)	(−0.28)	(0.30)	(−0.08)	(0.36)
Expectations		0.890^***^		0.609^***^		0.720^***^
		(20.47)		(7.40)		(13.42)
Constant	4.675^***^	0.424	4.271^***^	1.541^**^	4.384^***^	1.107^***^
	(9.07)	(1.56)	(9.70)	(3.20)	(13.23)	(3.48)
Observations	144	144	192	192	336	336
Cluster/Subjects	72	72	96	96	168	168
Adjusted *R*^2^	0.085	0.702	0.088	0.391	0.084	0.506

t-statistics in parentheses, OLS regression analysis used, standard errors are clustered at the individual level, variables group affiliation and round centered,

+ p < 0.10,

* p < 0.05,

** p < 0.01,

*** p < 0.001.

When analyzing cooperation in round 10 and 11 separately there was even a slightly lower cooperation rate toward the ingroup as compared to the outgroup in round 10, *b* = −1.25, *t*_(69)_ = −1.41, *p* = 0.16 and strong ingroup favoritism was observed in the first interaction with the new group *b* = 2.35, *t*_(69)_ = 3.21, *p* = 0.002. This is also illustrated in Figure 1 by the jump from a negative bias score in round 10 to a positive bias score in round 11. Hence, the significantly increasing bias score over several rounds is not a result of a gradual slope, but rather of an abrupt rise of ingroup favoritism from round 10 to round 11, where the group change and restart took place. Within the two phases of the experiment, the regression lines are rather flat or even decreasing (Figure 1, left). Running the same regression as before with the bias score as a criterion and adding a dummy for the experimental phase (before or after group change), the effect of round on ingroup favoritism is no longer significant.

Interestingly, when controlling for expectations, the group change effect is no longer significant either, indicating that changes in expectations mediate the effects of activated social identity due to group change (Table 4, Model 2). To further test whether the group change effect could partially be explained by expectations, we conduct a mediation analysis clustering across individuals and using bootstrapping to estimate standard errors (Preacher and Hayes, 2008). This analysis reveals a significant mediation [total indirect effect: *b* = 3.26, CI_95_:(0.97; 5.49)].^10^

### Discussion

In Experiment 1, we find ingroup favoritism to be a dynamic phenomenon that develops over contacts with the ingroup and the outgroup. This dynamic ingroup-favoring effect is not driven by differences in generalizing experiences or gradual changes over rounds. In contrast, we find that differences are mainly driven by one specific event, namely the change of groups that occurred after round 10. This finding can be due to the fact that a group change activates social identity by making the distinction between outgroup and ingroup more salient.

However, before rejecting the ingroup-favoring generalization bias as a potential influence factor, one has to point to several potential limitations of our experiment. First, it has to be acknowledged that the power of the experiment was limited due to the relatively low number of participants. Second, in one of the two labs involved (the lab in the city of Erfurt), mainly 1st year students (95%) took part in the experiment, who might still have had low identification with their university, perceived lower status or other characteristics that were not observable.

With our second experiment, we aimed to overcome these limitations and to test the stability of the findings more generally. To increase the chances of observing rational updating or generalization biases from the beginning, we made social identity more salient from the beginning by revealing to participants that they would interact with different people from the University of Bonn or the University of Erfurt. The resulting increase in group distinctiveness can be expected to affect group perceptions (Spears et al., 1985; Acorn et al., 1988; McConnell et al., 1994) and ingroup favoritism should generally increase with salience of the ingroup (see the meta-analysis by Mullen et al., 1992).

## Experiment 2

### Methods

Ninety-six people (mainly students from the University of Bonn and the University of Erfurt, 65 of whom were female) were recruited in the same way as in Experiment 1. Participants' payments depended on their decisions, and earnings ranged from 6.70 to 21.70 Euros (approx. USD 9.00 to USD 29.30). We applied the same procedure as in Experiment 1, with the exception that this time we explicitly named the city of the interaction partner in the instructions so that all participants were aware of whether they were interacting with students from the University of Bonn or Erfurt. Everything else remained the same.

### Results and discussion

Again, the group manipulation proved to be successful in that people indicated a higher interpersonal closeness between self and ingroup (*M* = 5.25, *SD* = 1.63), as compared to self and outgroup (*M* = 3.46, *SD* = 1.73), prior to playing the prisoner's dilemma game, *t*_(95)_ = 9.75, *p* < 0.001, *d* = 1.06.

Using the same analyses as in Experiment 1, we again find that cooperation is higher for the ingroup as compared to the outgroup, *b* = 1.01, *t*_(95)_ = 4.93, *p* < 0.001, *d* = 0.28 and the same holds for expectations, *b* = 1.15, *t*_(95)_ = 5.58, *p* < 0.001, *d* = 0.36 (Table 1, Models 3 and 4). Similar to Experiment 1, the bias score indicating average ingroup favoritism in the respective round increases over the course of time, *b* = 0.11, *t*_(18)_ = 3.90, *p* = 0.001, again speaking for the ingroup favoritism to be dynamic. Hence, in each of the 20 rounds, favoring the ingroup over the outgroup increases by 0.11 Taler. Furthermore, we replicate the effect that there is no significant difference in cooperation in the first round, *b* = 0.69, *t*_(93)_ = 0.94, *p* = 0.347 (Table 2, Model 3), but it appears in the last round, *b* = 1.83, *t*_(93)_ = 2.84, *p* = 0.005 (Table 2, Model 4).

We again do not find support for the ingroup-favoring generalization bias (Table 3, Models 3 and 4). Generalization of positive and negative experiences does not differ between ingroup and outgroup (Figure 2, right), with both respective interactions being reversed in direction and not significant. The strong effect of the change in groups after round 10, observed in Experiment 1, is not fully replicated, but a tendency in the same direction is observed, *b* = 1.04, *t*_(95)_ = 0.88, *p* = 0.383 (Table 4, Model 3; see also Figure 1, right). We have no conclusive explanation for why the magnitude of the effect is reduced but perhaps the stronger and more salient group manipulation that was applied already in the beginning of Experiment 2 might have contributed to it in that it reduced potential later contrast effects between ingroup and outgroup. As before, none of the comparisons between round 1 and 10 as well as between round 11 and 20 reveals significant changes. When running a regression predicting the bias score by round and adding a dummy for the experimental phase (before or after group change), the dynamic in ingroup favoritism is no longer significant, which is in line with the results of Experiment 1.

Experiment 2 was designed to readdress the development of ingroup favoritism with a more salient initial group manipulation at the beginning. We were thus able to replicate the result of a developing ingroup-favoring effect over repeated interactions in a prisoner's dilemma game. Again there was no support for the second hypothesis concerning systematic biases in generalization of experiences.

### Overall analysis

**Figure 3:**
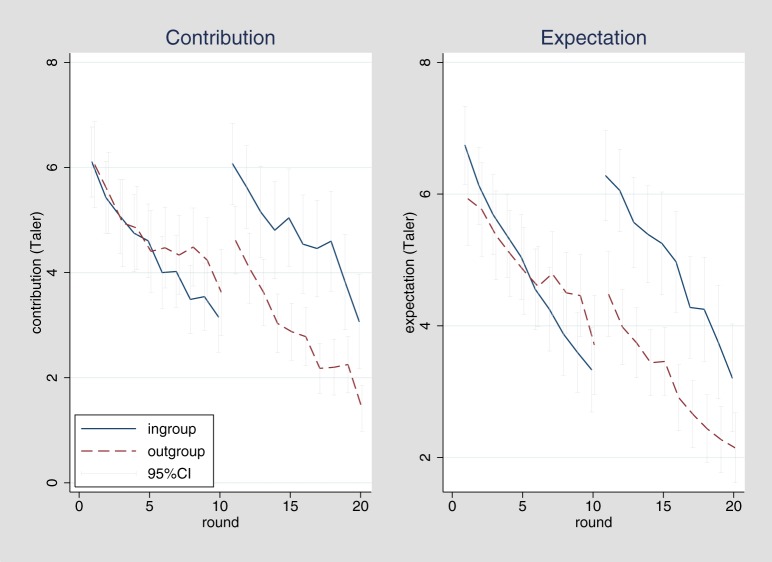
**Overall development of contributions and expectations in rounds 1–20**.

Given the similarity between Experiments 1 and 2, we conduct an overall analysis to generate best estimates concerning the dynamic effects.^11^ The detailed development of cooperation and expectations is shown in Figure 3.

**Figure 4:**
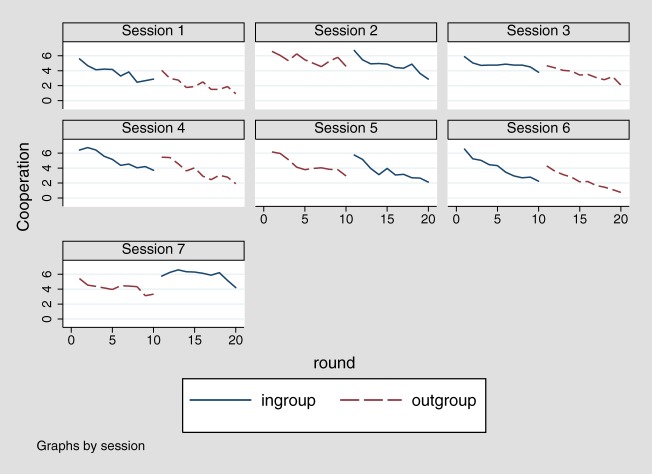
**Development of contributions per experimental session in rounds 1–20**.

Generally, there is clear evidence for a dynamic development of ingroup favoritism in cooperation since we find that ingroup favoritism (as indicated by the round-specific bias score) significantly increases over time, *b* = 0.15, *t*_(18)_ = 5.46, *p* < 0.001. There is no indication for a generalization bias in updating of expectations neither for positive, nor for negative experiences, although there is a strong effect of experience on updating in general (Table 3, Models 5 and 6). In the overall analysis, the effect of the change in groups between round 10 and 11 seems to be the main force driving differences between ingroup and outgroup (Table 4, Model 5). When controlling for expectations, the group change effect is no longer significant, again indicating that the effects of group change on ingroup favoring are mediated by expectations (Table 4, Model 6), which is further confirmed by a mediation analysis using bootstrapping to estimate cluster corrected standard errors (clustering at the level of individuals), *b* = 1.56, CI_95_:(0.46; 2.64). In contrast, ingroup favoritism does not change over repeated interactions with several members from the ingroup or the outgroup, as indicated by the fact that there are no systematic differences between rounds 1 and 10, *b* = −0.52, *t*_(167)_ = −0.82, *p* = 0.415, as well as rounds 11 and 20, *b* = 0.20, *t*_(167)_ = 0.34, *p* = 0.736. Overall, this result indicates that the dynamics we have observed are mainly due to changing groups, which makes the comparison between groups more salient. Gradual effects of biased generalization in repeated interactions are not observed and findings after the first interaction with a member from the respective group are more in line with rational models of belief updating that do not assume differential effects between ingroups and outgroups. A detailed development of cooperation by experimental session is shown in Figure 4. Interestingly, the comparison between sessions also reveals that effect of group change is larger when switching from outgroup to ingroup than vice versa.

Expectations (Figure 3, right) show a similar general pattern, although there is a tendency toward expecting more cooperation from the ingroup already in the first round, *b* = 0.81, *t*_(167)_ = 1.71, *p* = 0.089, which diminishes and even reverses in subsequent interactions with members from the same group up to round 10, *b* = −1.18, *t*_(167)_ = −1.82, *p* = 0.071. No systematic differences can be observed between rounds 11 and 20, *b* = −0.74, *t*_(167)_ = −1.12, *p* = 0.226.

## General discussion

A recent meta-analysis (Balliet et al., 2014) showed that, aggregated over the large set of available studies, individuals tend to cooperate more with members of their ingroup as compared to members of an outgroup. Overall, this effect of ingroup favoritism is small to medium in size and a similar difference prevails concerning expectations. The meta-analysis identified several moderators for ingroup favoritism. Of particular interest for our study was the finding that cooperation between the ingroup and the outgroup is stronger in repeated interactions with changing interaction partners from the same group (i.e., the ingroup or the outgroup), as compared to one-shot interactions. We expected that differences are due to dynamic developments over repeated interactions. To test this assumption, we assessed ingroup favoritism through cooperation behavior in a repeated continuous prisoner's dilemma where participants sequentially interacted with 10 members of the ingroup (own city and university) and subsequently with 10 different members of the outgroup (other city and university) or vice versa. Aggregated over all trials, we replicated ingroup favoritism in cooperation and expectation and found effects that are comparable in size to the results from the meta-analysis. More importantly, we observed a development of ingroup favoritism over time, in that the intergroup bias—the systematic tendency to evaluate the own group more positively or behave more positively toward the ingroup—in cooperation changed in favor of the ingroup over repeated contact with both groups. However, rational updating of expectations based on real differences in experienced cooperation with the ingroup as compared to the outgroup did not seem to be the crucial driver for the observed dynamics. We also did not find support for an ingroup-favoring generalization bias suggesting that people generalize differently (and in an ingroup-favoring way) over past experiences with ingroup or outgroup members. Although individuals updated their expectations concerning the behavior of members from the ingroup and the outgroup with repeated interactions, this updating process did not differ between groups and is hence unbiased with regard to the difference between ingroup and outgroup. Rather, we see that ingroup favoritism only occurred from the moment people effectively got into contact with the second of both groups, that is, after the group change in round 11. As illustrated in Figure 3, a systematic difference in restart effects can be observed. While restart effects in general are typical for behavior in repeated social dilemmas after a restart of the game (Cookson, 2000; Fischbacher et al., 2001), in our experiments the effect was particularly pronounced when playing with the outgroup first before interacting with the ingroup. The cooperation pattern during the first 10 rounds shows the typical declining pattern for social dilemma games. One could assume that, when contrasting both groups, people start more optimistically when first playing with an outgroup member followed by interactions with ingroup members (“my group will be much nicer than the other group”) as compared to the other way around (“the other group cannot be much better than my group”). When comparing our findings to the results from previous studies, it is worth noting that the meta-analysis (Balliet et al., 2014) indicates consistent ingroup favoring for one-shot interactions, while over two studies we consistently do not find ingroup favoring in the first round of a repeated interaction. Hence, the anticipation of subsequent interactions even with other persons seems to influence behavior.

Interestingly, expectations mediate the occurrence of ingroup favoritism after group change. The development of ingroup favoritism between round 10 and 11 (group change) is conveyed by increasing expectations when the interaction partner is from the ingroup compared to the outgroup. When controlling for expectations, the effect of group change on ingroup favoritism disappears.

Given that our result concerning the factors driving dynamics in ingroup favoritism leads to different results than a priori expected, and since Experiment 2 replicated the jump after group change only as a tendency, more studies are needed to validate our findings and conclusions further. We think that the research paradigm developed for this study is useful for this purpose, since it allows us to conduct investigations in a highly controlled setting. Although we did not find evidence supporting the ingroup-favoring generalization bias in our experiments, we would not exclude the possibility that such patterns might occur in other settings with more homogeneous groups or less anonymous intergroup contact. Additionally, strictly speaking we only have 3 (Experiment 1) and 4 (Experiment 2) independent observations, since all participants in one session were connected. Further studies with more observations would be recommended. However, there are natural limitations for running more subjects since the organization of such experiments in two labs that require full participation is rather cumbersome.

The present studies assess the development of ingroup favoritism, which is one aspect of parochial altruism. Parochial altruism explains intergroup conflict through two phenomena that have been closely linked in human evolution: the readiness to benefit the ingroup (ingroup love) and to harm the outgroup (outgroup hate). The prisoner's dilemma used in the current investigation allows one to study ingroup love elaborately. At the same time, the prisoner's dilemma does not allow one to assess people's motivation to harm the outgroup. Future studies should rely on extended paradigms that allow one to measure both components of parochial altruism separately (De Dreu, 2010) and to identify potential dynamics in both aspects.

### Conflict of interest statement

The authors declare that the research was conducted in the absence of any commercial or financial relationships that could be construed as a potential conflict of interest.

## Footnotes

^1^Due to technical problems, we lost one session, which led to an unbalanced number of order conditions (ingroup first vs. outgroup first). In two sessions (*N* = 48), participants interacted with the ingroup first; in one session (*N* = 24), participants interacted with the outgroup first.

^2^For Experiment 1, we also employed personality measures. Results for personality measures of Experiment 1 are not reported here (and were not collected for Experiment 2), but are available upon request. Instructions for both experiments were provided in German. An English translation is provided in the online supplementary materials.

^3^We assessed expectations prior to cooperation decisions and not the other way around since we were interested in the factors driving ingroup favoritism and therefore wanted to assess our predictor before our criterion. Due to the repeated interaction design, the order of expectation and action assessment is crucial only for the first round since afterwards in any case both kinds of assessments alternate and this was common knowledge already prior to the start of the game. Also, when analyzing the data without the very first round, the conclusion that ingroup favoritism is dynamic did not change.

^4^Participants completed the whole interpersonal closeness measure (perceived overlap between self and ingroup, self and outgroup, and ingroup and outgroup) on three different occasions: Before the experiment started, after the first 10 rounds (before participants received instructions for part 2 where they learned with whom they would interact in the second part of the experiment), and in the end. At all stages and for both studies, participants stated a higher overlap between self and ingroup, as compared to self and outgroup. In both studies, the perceived overlap between ingroup and outgroup diminished from time 1 to time 3. For 13 participants, we have missing values for the overlap between self and outgroup, since some participants forgot to complete the reverse side of the questionnaire.

^5^Due to collinearity, we did not additionally include session dummies (we only had one session where participants interacted with the outgroup first). To test the robustness of our findings we additionally confirmed our analyses by applying fixed-effects panel regressions with ingroup (vs. outgroup) as the within-subjects (fixed-effects) variable when appropriate. This analysis led to the same conclusions.

^6^In the two regression analyses we predict cooperation by group (ingroup vs. outgroup) in round 1 and round 20, respectively, additionally controlling for experimental lab. We do not control for counterbalancing condition since this information is included in the variable group due to considering the first and the last trial only.

^7^Interestingly and although we do observe ingroup favoritism in both experiments, when analyzing the first 10 rounds of Experiment 1 separately, we find a tendency toward outgroup favoritism, *b* = −1.20, *t*(71) = −1.91, *p* = 0.060. In previous studies occasionally also outgroup favoritism has been found in situations in which the status of the ingroup was low as compared to the outgroup (e.g., Jost and Burgess, 2000). In our study particularly effects of perceived status differences between own and other universities cannot be fully precluded. Furthermore, since in Erfurt mainly first-year students (95%) took part in the experiment, one could assume additional effects of status or it might be the case that they did not yet identify with their fellow students and preferred the outgroup instead. Both explanations remain highly speculative and future research would be necessary to explore this issue further.

^8^We conducted a regression analysis in which the change in expectations (current expectation – expectation in the previous round) was predicted by the previous experience with a member from the current group (received cooperation – expected cooperation), a variable coding our ingroup–outgroup manipulation (both variables centered) and their interaction. We furthermore again included the control variables “lab” and “condition” and clustered at the individual level. Analyses were run separately for positive experiences (i.e., receiving more than expected) and negative experiences (i.e., receiving less than expected). A further full factorial analysis including the three-way interaction of positive and negative experiences led to the same conclusions.

^9^Both variables centered.

^10^91% of the total effect could be explained by the indirect effect.

^11^Due to the imbalance of order conditions (ingroup vs. outgroup first) between Experiments 1 and 2, random assignment to experimental conditions is no longer given when pooling data over both experiments. Therefore we cannot rule out potential confounds concerning our results. However, when including a dummy for experiment (1 vs. 2) in the regression models (predicting generalization bias, group change, and expectations) to control for potential differences between studies, we observe the same pattern of results.
