# Supplementary material for: The development of ingroup favoritism in repeated social dilemmas
Source: Front Psychol. 2015 Apr 28;6:476. doi: 10.3389/fpsyg.2015.00476 (PMC4411968; doi:10.3389/fpsyg.2015.00476)
Supplement: Supplementary file 1 [file DataSheet1.DOCX]

**Appendix**

Experimental Instructions

Thank you very much for participating in our experiment. Please read the description of the experiment carefully and thoroughly. For the *entire* duration of the experiment, it is very important that you do not communicate with other participants.

If there is anything you do not understand, please look at your instructions once again.

Should you still have questions, please signal us by hand. We will then approach you in order to answer your questions in person.

In this experiment, you can earn money. Your individual payment for the experiment depends on *your* decisions and those of *other* participants. You will receive your aggregated payment directly after the experiment.

During the experiment, you will interact with other participants randomly assigned to you. These participants make their decisions simultaneously with you. This experiment is being conducted in two experimental laboratories simultaneously.

The experiment consists of two parts. You hereby receive the instructions for the first part, consisting of *ten rounds*.

During these ten rounds, you will interact either with persons who are sitting in the same experimental laboratory and study at the same university as yourself, or with persons who are sitting together in another experimental laboratory and are studying at a different university.

In each of these ten rounds, you will play with a different player, you will never encounter any one person twice.

The alternating players will *all* be sitting either *in the same experimental laboratory* and studying at *your university*, or they will be sitting in *another experimental laboratory* and studying at a *different university*.

Prior to the start of these ten rounds, you will receive information whether or not you will interact with players sitting *here in this experimental laboratory* and studying at *your university*, or in *another experimental laboratory* and studying at a *different university*. However, neither during the experiment, nor afterwards will you be told who these persons are. The players you interact with will likewise know whether or not you are sitting in the same laboratory as them.

**Set-up of a round**

At the beginning of the experiment, it will be randomly determined whether or not you will interact with players sitting *here in this experimental laboratory* and studying at *your university*, or with players in *another experimental laboratory* and studying at a *different university*. The information, whether you will interact with the group of persons in this laboratory or with the other group, will be given to you prior to the first on-screen decision.

In each of these ten rounds, you will be *randomly* assigned a new player from the *same* group.

You yourself (*player A*) and *player B* must simultaneously make a similarly structured decision.

For each round, each player receives a round endowment of 10 Taler (1 Taler = 0.05€)

You now have the option of *transferring* any amount of this round endowment *to player B*. However, you can only transfer integral amounts, i.e. you can only choose *one* number from the ones given [0, 1, 2, 3, 4, 5, 6, 7, 8, 9, 10].

The amount you transfer to player B will be *doubled*, i.e. player B receives twice the amount you transferred,

The randomly assigned player B has exactly the same options. He also has the option of transferring an amount to you. Both players make their choices *simultaneously*.

During each round, you will be asked to estimate how many coins (0-10) player B will transfer to you.

*Screenshot of belief stage*

Afterwards, you can choose how many coins you want to transfer to the other player.

*Sreenshot of contribution stage*

At the end of each round, you will be informed about your earnings from that round.

*Screenshot round results*

Your payout is determined as follows:

| Your round endowment |
| --- |
| - the amount you have transferred to player B |
| + twice the amount that player B has transferred to you |
| = your individual payout |

**Comprehension questions**

1. What is your income after the first round, if you transfer *10 Taler* to player B and player B likewise transfers *10 Taler* to you?
2. What is your income after the first round, if you transfer *0 Taler* to player B and player B transfers *10 Taler* to you?

_____

1. What is your income after the first round, if you transfer *10 Taler* to player B and player B transfers *0 Taler* to you?

_____

1. What is your income after round 5, if you start round 5 with a previous income of *50 Taler* plus the round endowment of *10 Taler,* and you transfer *8 Taler* of these to player B and player B transfers *2 Taler* to you?

_____

1. What is the the income of player B after the first round, if you transfer *8 Taler* to player B and player B transfers *2 Taler* to you?

_____

1. With what transfer amount could you and player B maximize the collective income in one round (i.e. the sum of incomes) for both of you?

______

Please signal us by hand once you have answered these questions.
